# Association between excessive maternal weight, periodontitis during the third trimester of pregnancy, and infants’ health at birth

**DOI:** 10.1590/1678-7757-2019-0351

**Published:** 2020-03-27

**Authors:** Gerson Aparecido FORATORI-JUNIOR, Bruno Gualtieri JESUINO, Rafaela Aparecida CARACHO, Eliel Soares ORENHA, Francisco Carlos GROPPO, Silvia Helena de Carvalho SALES-PERES

**Affiliations:** 1 Universidade de São Paulo Faculdade de Odontologia de Bauru Departamento de Odontopediatria, Ortodontia e Saúde Coletiva BauruSão Paulo Brasil Universidade de São Paulo, Faculdade de Odontologia de Bauru, Departamento de Odontopediatria, Ortodontia e Saúde Coletiva, Bauru, São Paulo, Brasil.; 2 Universidade Estadual de Campinas Faculdade de Odontologia de Piracicaba Departamento de Ciências Fisiológicas, área de Farmacologia PiracicabaSão Paulo Brasil Universidade Estadual de Campinas, Faculdade de Odontologia de Piracicaba, Departamento de Ciências Fisiológicas, área de Farmacologia, Piracicaba, São Paulo, Brasil.

**Keywords:** Pregnancy, Obesity, Overweight, Periodontitis, Birth weight

## Abstract

Excessive weight is associated with periodontitis because of inflammatory mediators secreted by the adipose tissue. Periodontal impairments can occur during pregnancy due to association between high hormonal levels and inadequate oral hygiene. Moreover, periodontitis and excessive weight during pregnancy can negatively affect an infant’s weight at birth.

**Objective:**

This observational, cross-sectional study aimed to evaluate the association between pre-pregnancy overweight/obesity, periodontitis during the third trimester of pregnancy, and the infants' birth weight.

**Methodology:**

The sample set was divided into 2 groups according to the preconception body mass index: obesity/overweight (G1=50) and normal weight (G2=50). Educational level, monthly household income, and systemic impairments during pregnancy were assessed. Pocket probing depth (PPD) and clinical attachment level (CAL) were obtained to analyze periodontitis. The children’s birth weight was classified as low (<2.5 kg), insufficient (2.5–2.999 kg), normal (3–3.999 kg), or excessive (≥4 kg). Bivariate analysis (Mann-Whitney U test, t-test, chi-squared test) and logistic regression (stepwise backward likelihood ratio) were performed (p<0.05).

**Results:**

G1 showed lower socioeconomic levels and higher prevalence of arterial hypertension and gestational diabetes mellitus during pregnancy than G2 (p=0.002). G1 showed higher means of PPD and CAL (p=0.041 and p=0.039, respectively) and therefore a higher prevalence of periodontitis than G2 (p=0.0003). G1 showed lower infants’ birth weight than G2 (p=0.0004). Excessive maternal weight and educational levels were independent variables associated with periodontitis during the third trimester of pregnancy (X^2^[2]=23.21; p<0.0001). Maternal overweight/obesity was also associated with low/insufficient birth weight (X^2^[1]=7.01; p=0.008).

**Conclusion:**

The present findings suggest an association between excessive pre-pregnancy weight, maternal periodontitis, and low/insufficient birth weight.

## Introduction

Patients with obesity have high levels of pro-inflammatory adipokines and cytokines that negatively affect the individuals’ immunity, thus increasing the inflammatory response. The adipose tissue secretes inflammatory mediators that cause a widespread inflammatory state in the body of the obese patients. As a result, these patients may have a significant inflammatory response in the periodontal tissues, even in the presence of a normal amount of dental plaque.^1^

Pregnancy can also negatively affect a patient’s oral condition. During pregnancy hormonal alterations cause physiological and anatomical changes. These changes occur from conception to labor and promote fetal growth and pregnancy maintenance. High levels of progesterone and estrogen cause oral alterations during pregnancy decreasing an individual’s immune response.^2^Therefore, with the tooth biofilm formation, the patient’s periodontal condition can worsen during this period.

Studies have reported a triangular association between obesity/overweight and periodontal status during pregnancy.^3-10^ All studies found a positive association between obesity/overweight and periodontal status; however, they did not use the same classifications for periodontitis and body mass index (BMI), and none of the studies analyzed the association of these factors with infants’ health at birth.

The scientific literature highlights the association between periodontitis and adverse delivery outcomes, such as preterm birth and low birth weight.^11-15^ In contrast, maternal overweight/obesity is strongly associated with insulin resistance, which, in turn, is related to the gestational diabetes mellitus (GDM), preeclampsia, and macrosomia.^16-19^ However, studies that considered maternal overweight/obesity, periodontitis, and birth weight have not been conducted yet. Therefore, this study aimed to evaluate the association between pre-pregnancy overweight/obesity, periodontitis during the third trimester of pregnancy, and birth weight.

## Methodology

The Strengthening the Reporting of Observational studies in Epidemiology (STROBE) guidelines were used to ensure the accurate reporting of this observational, cross-sectional study.^20^

### Ethical approval

According to the Declaration of Helsinki, this study was approved by the Ethics Committee on Human Research (CAAE 58339416.4.0000.5417). All participants provided written informed consent.

### Sample conformation

One hundred women in the third trimester of pregnancy were included in this study and subsequently divided into 2 groups according to preconception BMI: pregnant women with obesity or overweight (G1=50) and pregnant women with normal weight (G2=50). The third trimester of the pregnancy was chosen to be evaluated in this study because of its high hormonal levels.^2^ The participants were recruited from a Brazilian public healthcare system in Bauru City, São Paulo from August 2017 to November 2018. The pre-pregnancy BMI was considered because the main objective of this study was to analyze how overweight/obesity could affect the systemic and oral health during pregnancy. For pre-pregnancy BMI calculations, pre-pregnancy weights were collected from medical follow-up. Heights were obtained during the appointment using a stadiometer (Wood 2.20, WCS Ind.; Curitiba, Paraná, Brazil). A normal pre-pregnancy weight was defined as a BMI of 18.0–24.99 kg/m^2^, while pre-pregnancy overweight was defined as a BMI between 25.0 and 29.99 kg/m^2^ and pre-pregnancy obesity as BMI ≥30.0 kg/m^2^.^21^The patient’s weight during the third trimester of pregnancy was recorded to evaluate their BMI during this period.

The inclusion criteria were: women in the third trimester of pregnancy, women with satisfactory systemic health, and women undergoing regular medical visits. Patients with a BMI higher than 25 kg/m^2^ were assigned to G1 and those with a BMI from 18 to 24.99 kg/m^2^ were assigned to G2. The exclusion criteria were: patients with general health impairments; patients with diabetes mellitus and/or arterial hypertension (AH) before pregnancy; patients with a BMI lower than 18 kg/m^2^; patients with smoking, drug, or alcohol habits; patients with gestational impairments that required absolute rest; and/or patients who were taking medications that could negatively affect their oral health. Furthermore, twin or multiple pregnancies were excluded in this study. Patients who had their infants before the 37^th^ gestational week and after the 40^th^ gestational week were excluded from this study.

### General evaluations

Data regarding systemic impairments, such as diabetes mellitus and AH during pregnancy, were obtained from medical records and registered as 0 (absent) or 1 (present). AH in pregnancy was established as ≥140/90 mmHg blood pressure levels.^22^ The diagnosis of GDM was based on the International Association of the Diabetes and Pregnancy Study Group protocol,^23^ which defined the diagnosis of maternal hyperglycemia as having a fasting blood sugar level of ≥92 mg/dl. Educational level and monthly household income were classified according to a previous study by our research team.^4^ This study categorized the educational level along these lines: 0, illiteracy; level I, did not complete the primary education; level II, completed the primary education; level III, did not complete high school; level IV, completed high school; level V, did not complete higher education; level VI, completed higher education; level VII, specialization; level VIII, master’s degree; and level IX, Doctor of Philosophy (PhD). This study registered the highest educational level.

Monthly household income was graded into the following levels according to minimum wage (MW): Level I, up to 1 MW; Level II, 1–2 MW; Level III, 2–3 MW; Level IV, 3–4 MW; Level V, 4–5 MW; and Level VI, higher than 5 MW. The minimum wage considered in this study is approved by the Brazilian government as R$ 937.00 (approximately USD 284.00). Moreover, oral hygiene behaviors were assessed such as daily frequency of toothbrushing and use of daily dental floss.

### Clinical periodontal assessment

A calibrated dentist performed oral assessments to assess the periodontal condition (kappa intra-examiner=0.95; 95% confidence interval [CI]=0.89–0.97; kappa inter-examiner=0.92; 95% CI=0.87–0.95). Before sample recruitment, calibration of the examiner for the periodontal parameters was performed in 10 patients , corresponding to 10% of the total sample. These 10 patients were not included in this study. The periodontal measurements were obtained at different times by a primary examiner and by a gold-standard investigator to obtain the intra- and inter-examiner concordance coefficient, considering the periodontal fiber changes after the first periodontal probing. All teeth present in the mouth were evaluated, excluding the third molars. The periodontal assessment was performed according to the pocket probing depth (PPD) and the clinical attachment level (CAL). The PPD was measured from the free gingival margin to the bottom of the periodontal pocket and the CAL was measured from the cementoenamel junction to the base of the periodontal pocket.^4^ Gingival recession and hyperplasia were also measured to calculate the CAL. Six sites of each tooth were assessed: mesial, center, distal, both buccal, and lingual.^4^ The mean values for the PPD and the CAL were obtained. Periodontitis was diagnosed when the patients had ≥2 interproximal sites with CAL ≥3 mm and ≥2 interproximal sites with a PPD≥ 4 mm (not on the same tooth) or one site with a PD ≥5 mm, according to case definitions for surveillance of periodontitis proposed by Page and Eke^24^ (2007). Furthermore, periodontal disease was categorized as moderate and severe.^24^

### Birth weight at birth analysis

**Figure 1:**
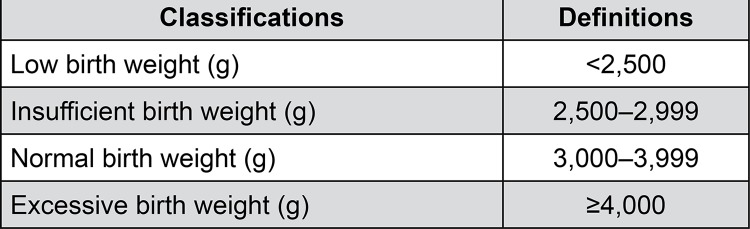
Definitions for infants’ weight at birth g= grams

The children’s weight at birth was provided by the patients after the delivery and classified according to the World Health Organization (WHO) into low/insufficient, normal, and excessive (Figure 1).^25-27^

### Statistical analysis

Statistical analysis was performed using the International Business Machines Corporation Statistical Package for the Social Sciences (IBM SPSS) version 25 (IBM Corp.; Armonk, New York, United States). Once we ensured that 10 patients were selected for each combination of independent variables, the protocol proposed by Hosmer and Lemeshow for regression logistic analysis was adapted regarding sample size^28^. Therefore, initially, the dichotomization of periodontitis (0, no periodontitis; 1, with periodontitis) and low/insufficient birth weight (0, no low/insufficient birth weight; 1, low/insufficient birth weight) was performed, followed by an inclusion of a maximum of 5 independent variables in the logistic models. Thus, a sample size of 100 was considered representative. The Kolmogorov-Smirnov test was applied to verify the normal distribution of the sample. The t-test was used in bivariate analysis to examine the normally distributed quantitative variables. The Mann-Whitney U test was used to examine non-normally distributed quantitative variables and ordinal variables. The chi-squared test was used to examine binomial data. The logistic regression was adapted using the stepwise backward (likelihood ratio) method to analyze the independent variables associated with periodontitis and infants’ birth weight (dependent variables). A significance level of 5% was considered statistically significant. Hosmer-Lemeshow, collinearity, and residual analyses were used to increase our understanding of the logistic regression results.

## Results

Initially, 123 patients were selected for this study; out of these, 23 were excluded due to the following reasons: requiring absolute rest (n=3), history of periodontitis before pregnancy (n=2), more than 40 weeks of gestation (n=9), smokers (n=2), underweight (n=3), and under orthodontic treatment (n=4). Therefore, 100 patients were included in this study.

The participants’ average age was 29.5 years (30.4 and 28.6 years in G1 and G2, respectively). G1 had lower family income (p=0.011) and lower educational level than G2 (p=0.034).G1 also had a lower prevalence of patients who had completed higher education (n=19; 38%) compared with G2 (n=27; 54%) (Table 1).

**Table 1:** Comparison between the groups regarding contextual, periodontal, and systemic characteristics

Characteristics	G1 (n = 50)	G2 (n = 50)	p
	Mean ± SD	Mean ± SD	
	Median [1st-3rd quartiles]	Median [1st-3rd quartiles]	
Maternal age (years)	30.5 [28 - 33]	28.5 [23 -33]	0.144^†^
Education level	4 [3 - 6]	5 [4 - 6]	0.034^†^
Household monthly income	3 [2 - 5]	5 [3 - 6]	0.011^†^
Pre-pregnancy BMI (kg/m^2^)	28.30 [26.56 - 33.33]	22.62 [20.79 - 24.09]	<0.0001^†^
BMI – 3rd trimester (kg/m^2^)	31.63 [29.88 - 35.48]	25.39 [23.38-28.05]	<0.0001^†^
Daily toothbrushing	3 [3 - 3]	3 [2 - 3]	0.757^†^
Daily dental floss use	1 [0 - 1]	1 [0 - 1]	0.392^†^
PPD mean (mm)	2.18 ± 0.38	2.03 ± 0.31	0.041*
CAL mean (mm)	2.16 [1.96 - 2.41]	2.03 [1.88 - 2.29]	0.039*
Periodontitis, n (%)			0.0003^‡^
No	14 (28%)	32 (64%)	
Yes	36 (72%)	18 (36%)	
Children’s birth weight (kg)	3.06 ± 0.35	3.33 ± 0.38	0.0004*
Low/insufficient weight at birth, n (%)			0.0008^‡^
No	32 (64%)	46 (92%)	
Yes	18 (36%)	4 (8%)	
AH during pregnancy, n (%)			0.002^‡^
No	37 (74%)	48 (96%)	
Yes	13 (26%)	2 (4%)	
GDM, n (%)			0.002^‡^
No	37 (74%)	48 (96%)	
Yes	13 (26%)	2 (4%)	

p= significance level; SD= standard deviation; * T test; ^†^ Mann-Whitney U test; ^‡^ Chi-square test

G1 showed a higher pre-pregnancy and gestational BMI than G2 (p<0.0001). Furthermore, G1 had a higher prevalence of hypertension and GDM than that of G2 (p=0.002) (Table 1).

The groups did not differ regarding daily toothbrushing frequency (p=0.757) and daily use of dental floss (p=0.392). However, they differed in the mean PPD (p=0.041), in the mean CAL (p=0.049), and in the prevalence of periodontitis, which was higher in G1 than in G2 (p=0.0003), with 48% (n=24) and 24% (n=12) of patients classified as having moderate and severe periodontitis respectively. None of the patients in G2 were classified as having severe periodontitis, but 36% (n=18) of the patients were classified as having moderate periodontitis (Table 1).

The average birth weights for G1 and G2 were 3.06±0.35 kg and 3.33±0.38 kg, respectively. No infants were classified as having excessive birth weight. However, 18 infants in G1 (36%) had a low or insufficient weight, whereas only 4 infants in G2 (8%) had a low or insufficient weight (p=0.0008) (Table 1).

Binary logistic regression was applied to determine the independent variables related to periodontitis (0, no periodontitis, 1, with periodontitis) during the third trimester of pregnancy (Table 2). Pre-pregnancy BMI, educational level, and AH and diabetes mellitus during pregnancy were variables inserted into the model. The multicollinearity analysis showed all independent variables showed values of tolerance greater than 0.10 and variance inflation factor (VIF) values lower than 2. The pre-pregnancy BMI and educational level were the variables of the final logistic model (X^2^[2]=23.21; p<0.0001; R^2^ of Nagelkerke=0.276). The overall percentage accuracy of the final model was 65%. The Hosmer and Lemeshow analysis indicated a chi-squared value for the final model of 2.58 for 8 degrees of freedom (p=0.958). Maternal BMI (adjusted odds ratio [OR]=1.12, 95% CI=1.02–1.21, p=0.024) and educational level (adjusted OR=0.642, 95% CI=0.489–0.842, p=0.001) were significant predictors of periodontitis in the third trimester of pregnancy.

**Table 2:** Binary logistic regression model showing the independent variables associated with periodontitis during the third trimester of pregnancy

Models	Variables	Adj. OR	95% CI		p
Model 1	Maternal BMI	1.11	1.00	1.22	0.054
	Education level	0.06	0.48	0.83	0.001
	AH during pregnancy	0.76	0.17	3.40	0.730
	GDM	1.21	0.29	5.11	0.786
	Constant	0.63			0.826
Model 2	Maternal BMI	1.11	1.00	1.21	0.054
	Education level	0.63	0.48	0.83	0.001
	AH during pregnancy	0.76	0.17	3.41	0.730
	Constant	0.79			0.904
Model 3	Maternal BMI	1.12	1.01	1.21	0.024
	Education level	0.64	0.49	0.84	0.001
	Constant	0.51			0.635

BMI= body mass index; AH= arterial hypertension; GDM= gestational diabetes mellitus; Adj. OR= adjusted odds ratio; CI= confidence interval; p= significance level

Moreover, the binary logistic regression was performed to determine the independent predictive variables of insufficient birth weight (0, normal birth weight; 1, low/insufficient birth weight) (Table 3). The variables inserted into the model were the following: pre-pregnancy BMI, educational level, presence of periodontitis, and AH and diabetes mellitus during pregnancy. The multicollinearity analysis showed that all independent variables indicated tolerance values greater than 0.10 and the VIF values lower than 2. Maternal BMI was the variable of the final logistic model (X^2^[1]=7.01; p=0.008; R^2^ of Nagelkerke=0.104) related to insufficient birth weight. The overall percentage accuracy of the final model was 77%. The Hosmer and Lemeshow analysis established a chi-squared value for the final model of 19.78 for 8 degrees of freedom (p=0.011). The maternal BMI (adjusted OR=1.11, 95% CI=1.02–1.19, p=0.012) was a significant predictor of insufficient birth weight.

**Table 3:** Binary logistic regression model showing the independent variables associated with low/insufficient infants’ weight at birth

Models	Variables	Adj. OR	95% CI		p
Model 1	Maternal BMI	1.10	1.00	1.19	0.061
	Education level	0.98	0.73	1.32	0.934
	AH during pregnancy	0.65	0.16	2.69	0.561
	GDM	2.57	0.49	13.23	0.259
	Periodontitis	0.49	0.16	1.53	0.224
	Constant	0.02			0.003
Model 2	Maternal BMI	1.10	1.00	1.19	0.060
	AH during pregnancy	0.66	0.16	2.68	0.564
	GDM	2.52	0.51	12.30	0.252
	Periodontitis	0.49	0.16	1.43	0.192
	Constant	0.02			0.051
Model 3	Maternal BMI	1.11	1.02	1.20	0.019
	DMG	2.53	0.52	12.37	0.251
	Periodontitis	0.49	0.17	1.43	0.195
	Constant	0.01			0.005
Model 4	Maternal BMI	1.09	1.00	1.17	0.038
	Periodontitis	0.52	0.18	1.50	0.226
	Constant	0.03			0.007
Model 5	Maternal BMI	1.11	1.02	1.19	0.012
	Constant	0.02			<0.001

BMI= body mass index; AH= arterial hypertension; GDM= gestational diabetes mellitus; Adj. OR= adjusted odds ratio; CI= confidence interval; p= significance level

## Discussion

This study contributes to the body of scientific evidence by evaluating the association between pre-pregnancy overweight/obesity, maternal periodontitis, and low birth weight. We adapted the standardized protocols for nutritional status classification and periodontitis diagnosis. The main findings of this study suggest pre-pregnancy excessive weight is significantly associated with periodontitis during the third trimester of pregnancy. Moreover, overweight/obesity is associated with a low/insufficient birth weight.

Obesity is a chronic and multifactorial disease and an important risk factor for type 2 diabetes, hypertension, coronary heart disease, osteoarthritis, and metabolic syndrome.^29^In this study, the overweight/obese pregnant women had a higher prevalence of AH and GMD than that of non-overweight/nonobese pregnant women (p=0.022). The presence of adiposity in the body was associated with the visceral accumulation of the adipose tissue, which directly contributes to insulin resistance and becomes more evident during pregnancy.^30^ Hypertension, in turn, is related to the vascular inflammation and endothelial disturbance. The vascular inflammation involves the release of inflammatory cytokines and adipokines that increase the vascular permeability and promote cytoskeletal changes in the endothelial cells, which can disrupt the balance between vasodilation and vasoconstriction. Inflammatory mediators secreted by the patients’ adipose tissue are also involved in this process and in the exacerbate vasoconstriction, causing an increase in blood pressure.^3^

A lower socioeconomic level has previously been shown to be associated with periodontitis and obesity.^4,8,31^ In this study, pregnant women with excessive weight had lower educational levels (p=0.034) and lower monthly household incomes (p=0.011) compared with pregnant women with non-excessive weight (Table 1). This can be explained by the fact that individuals with lower socioeconomic status show inadequate eating behaviors because they are reliant on cheaper foods that have more calories and lower nutritive content, resulting in overweight and obesity.^4,8,31^ The association between periodontitis and lower socioeconomic levels exists because the patients who have both conditions have lesser access to healthcare services and lesser access to oral hygiene education programs.^8^ However, in this study, the groups did not differ in the daily toothbrushing frequency or in the daily use of dental floss. Both the BMI increase and the lower educational levels were considered as independent variables of periodontitis according to the final logistic regression model (Table 2).

The association between obesity and periodontitis can be explained by the fact that the adipose tissue of the patients with excessive weight secretes inflammatory mediators, such as the tumor necrosis factor alpha, the interleukin 6, and the C-reactive protein, which can make the host more susceptible to inflammation.^1,4,32^ Therefore, patients with overweight/obesity can have higher levels of inflammation anddestruction of the periodontium, even in the presence of a normal amount of bacterial plaque, compared with patients with normal weight.

Periodontal impairments can also occur during pregnancy due to profound disturbances in the maternal immune system and increased levels of progesterone and estrogen. During pregnancy, hormonal disturbances increase the host’s susceptibility to oral inflammation even in the presence of a normal amount of bacterial dental plaque.^2,33^

Pre-pregnancy excessive weight, excessive gestational weight gain and GDM are associated with: infants large for gestational age, high birth weight, andmacrosomia.^16-19^ Pregnant women with GDM have a reduction in the peripheral insulin sensitivity, leading to maternal hyperglycemia, fetal hyperglycemia, and consequent hyperinsulinemia, resulting in excessive fetal growth.^34^ Obesity , in turn, has a significant effect on the macronutrient metabolism, altering glucose homeostasis, lipid oxidation, and amino acid synthesis, resulting in a possible interference with the fetus development.^35^

In contrast, women diagnosed with periodontitis during pregnancy are more likely to have preterm births and infants with low birth weight than women not diagnosed with periodontitis during pregnancy.^11-15,33,35^ Two pathogenic mechanisms are proposed to explain this association, known as the direct and the indirect pathways. The direct pathway is related to the presence of Gram-negative anaerobic bacteria originated in the gingival biofilm, whereas the indirect pathway involves the production of pro-inflammatory markers that enter the bloodstream from the gingival submucosa. Both mechanisms result in the development of an immune-mediated inflammatory response and/or local suppression of growth factors in the fetal-placental unit.^35^

The first study that suggested oral bacteria influenced the pregnancy outcomes was reported by Collins, et al.^36^ (1994). It highlighted that injecting pregnant hamsters with *Porphyromonas gingivalis* led to intrauterine growth retardation and to smaller fetuses, together with increased levels of pro-inflammatory mediators [interleukin 1 beta (IL-1b) and prostaglandin E2 (PGE2)] in the amniotic fluid.

However, studies that evaluate the association of these factors while considering the presence of periodontitis have not been conducted yet. In this study pregnant women with excessive weight had infants with low/insufficient birth weight (p=0.0008), in addition to the higher prevalence of periodontitis. Therefore, periodontitis seems to be significantly associated with infants’ health at birth. Nevertheless, according to the logistic regression related to infant’s weight at birth, overweight/obesity was the independent variable that persisted on the final logistic model, while the presence of periodontitis prevailed until model 4 of the logistic regression (p=0.226, Table 3). The authors hypothesize that periodontitis could be one of the variables to remain until the final logistic model in a future larger sample study. The findings of our study are in contrast with those that sought to assess the association between pre-pregnancy overweight/obesity and birth weight. It is hypothesized that inflammatory mediators (e.g., IL-1b and PGE2) are directly associated with low birth weight.^36^ The inflammatory mediators secreted by the adipose tissue in overweight individuals^1,32^ may also play a key role in the higher prevalence of underweight infants due to the indirect effect of the pro-inflammatory markers. Future prospective studies with larger samples must be conducted to better understand the association between maternal overweight/obesity, periodontitis, and birth weight based on this indirect effect. This study has some limitations. A longitudinal study with a larger sample size is required to further understand the association between maternal overweight/obesity, periodontitis, and birth weight. Additionally, this study considered obese and overweight patients in the same group. Future prospective studies are required to evaluate the effect of obesity only on periodontitis during pregnancy and the subsequent birth weight. Moreover, future studies should collect the participants’ visual dental plaque data to ensure the comparison between groups regarding the quality of oral hygiene habits.

Despite the limitations of this study, it is considered relevant to better understand the association between periodontitis, overweight/obesity during pregnancy, and low birth weight. This is notable as no previously published studies consider maternal systemic health, periodontal condition during pregnancy, and infants’ health at birth.

## Conclusion

These findings suggest an association between excessive pre-pregnancy weight, maternal periodontitis, and low/insufficient birth weight. Additionally, periodontitis seems to be associated with the patient’s socioeconomic status, and this should be considered during the comprehensive and multidisciplinary care of the overweight/obese pregnant women.
